# *Echinacea purpurea* (L.) Moench Polysaccharide Alleviates DSS-Induced Colitis in Rats by Restoring Th17/Treg Balance and Regulating Intestinal Flora

**DOI:** 10.3390/foods12234265

**Published:** 2023-11-25

**Authors:** Yaoxing Li, Yongshi Lin, Xirui Zheng, Xiaoman Zheng, Mingen Yan, Huiting Wang, Cui Liu

**Affiliations:** 1 College of Veterinary Medicine, South China Agricultural University, Guangzhou 510642, Chinazhengxr@stu.scau.edu.cn (X.Z.); zhengxm@stu.scau.edu.cn (X.Z.); yanmingen@stu.scau.edu.cn (M.Y.);; 2 Guangdong Technology Research Center for Traditional Chinese Veterinary Medicine and Nature Medicine, Guangzhou 510642, China; 3 International Institute of Traditional Chinese Veterinary Medicine, Guangzhou 510642, China

**Keywords:** ulcerative colitis, *Echinacea purpurea* (L.) Moench polysaccharide, Th17/Treg balance, TLR4/MyD88/NF-κB, intestinal flora

## Abstract

*Echinacea purpurea* is popularly used as a food supplement or nutritional supplement for its immune regulatory function against various threats. As one of its promising components, *Echinacea purpurea* (L.) Moench polysaccharide (EPP) has a wide range of biological activities. To evaluate the effect of EPP as a dietary supplement on ulcerative colitis (UC), this study used sodium dextran sulfate (DSS) to induce a UC model, extracted EPP using the ethanol subsiding method, and then supplemented with EPP by gavage for 7 days. Then, we evaluated the efficacy of EPP on DSS rats in terms of immunity, anti-inflammation, and intestinal flora. The result showed that EPP could alleviate colonic shortening and intestinal injury in rats with DSS-induced colitis, decrease the disease activity index (DAI) score, downregulate serum levels of inflammatory cytokines, and contribute to the restoration of the balance between the T helper cells 17 (Th17) and the regulatory T cells (Treg) in the spleen and mesenteric lymph nodes (MLNs). Meanwhile, EPP could downregulate the expression of Toll-like receptors 4 (TLR4), myeloid differentiation factor 88 (MyD88), and nuclear factor kappa-B (NF-κB) in colon tissue. In addition, the results of 16SrRNA sequencing showed that EPP also had a regulatory effect on intestinal flora of UC rats. These results indicate that EPP might achieve a beneficial effect on UC rats as a dietary supplement through restoring Th17/Treg balance, inhibiting the TLR4 signaling pathway and regulating intestinal flora, suggesting its possible application as a potential functional food ingredient alleviating UC.

## 1. Introduction

Ulcerative colitis (UC) is a form of inflammatory bowel disease (IBD) that primarily affects the colon, characterized by prolonged inflammation along with harm to the intestinal mucosa. Abdominal pain, blood in the stool, recurrent diarrhea, and depression are the basic symptoms of UC [1]. At present, it is commonly accepted that the etiology of UC may be influenced by interactions between the immune system, intestinal flora, environment, and host genetics [2]. However, the drugs available for UC are insufficient. For instance, 5-aminosalicylic acid and certain antibiotics commonly used in UC treatment have various limitations and side effects [3]. Therefore, it is of great significance to actively seek and develop new therapeutic drugs. 

T helper cells 17 (Th17) and regulatory T cells (Treg) are two functionally distinct subpopulations formed by the differentiation of activated CD4^+^ T cells [4]. Current evidence suggests the equilibrium of Th17 and Treg cells possess a crucial role in the development of UC. Th17 cells can produce pro-inflammatory cytokines, including interleukin-17 (IL-17), interleukin-22 (IL-22), and interleukin-23 (IL-23), while Treg cells can release transforming growth factor-β (TGF-β) and interleukin-10 (IL-10) to exert anti-inflammatory effects [5]. Correcting the immune imbalance caused by intestinal Th17/Treg dysregulation has become a new therapeutic approach for UC [6]. The intestinal flora is defined as all of the microorganisms that reside in or on the digestive tract [7]. The ability of cecum material to transmit behavioral abnormalities linked to intestinal inflammation has been proven [8]. Toll-like receptors (TLRs) act as bridges between immune response and intestinal microbiota. Certain microbial structures, such as lipopolysaccharides can be recognized by TLRs, whose activation can trigger cascade reactions, leading to an excess of inflammatory cytokines [9]. Furthermore, it has been demonstrated that the development of UC is often associated with dysregulation of the intestinal flora. The available data suggests that the human intestinal actinomyces *Eggerthella lenta* can induce intestinal Th17 activation by releasing the inhibition of retinoic-acid-receptor-related orphan nuclear receptor gamma (RORγt) [10]. Some bacteria associated with butyrate production, including *f. p auusnizzi* and *Clostridium perfringens* cluster IV, XIVa, XVIII, can influence the differentiation of Treg cells [11].

*Echinacea purpurea* (L.) Moench (EP) is a traditional herbal remedy native to North America. *Echinacea* spp. extracts and derivatives have been demonstrated to induce specific immune cell activities and are popularly used as food supplements or nutraceuticals with immuno-modulatory functions [12,13]. In modern pharmacological research, EP has been found to exhibit various biological activities, including immune regulation and anti-inflammatory and antioxidant effects [14]. The pharmacological activities of natural polysaccharides have received much attention in recent research [15]. Some studies indicated that *Echinacea purpurea* (L.) Moench polysaccharides (EPP) alleviated LPS-induced lung injury via inhibiting inflammation and apoptosis [16]. The effects of EPP on antioxidative stress and protection of the intestinal barrier have also been reported [17,18]. In a preliminary experimental trial, it was identified that there were more than 30 components in *Echinacea purpurea* (L.) Moench including caffeic acid, ferulic acid, vanillic acid, chicoric acid, quercetin, and others that may be involved in the treatment of ulcerative colitis [19]. And it was discovered that *Echinacea purpurea* (L.) Moench extract had a therapeutic effect on damp-heat diarrhea rats and could alleviate TNBS-induced colitis by regulating the complement system. Furthermore, both polysaccharides and sulfated modified polysaccharides in EP have significant immune enhancing effects on chicken and the predicted targets of EP for enhancing immune function, including signal transducer and activator of transcription 3 (STAT3) and toll-like receptors 4 (TLR4), were obtained through network pharmacology, which provided ideas and support for our experimental design [20,21].

The current study probed the efficacy of Echinacea polysaccharides on DSS-induced colitis in rats from the view of Th17/Treg immune homeostasis and regulation of intestinal flora. It was hoped that it could provide some support for the use of EPP as a food additive and a new drug option for the treatment of UC.

## 2. Materials and Methods

### 2.1. Plant Material, Chemicals and Regents

The whole plant of EP was provided by Guangdong Huanong Hi Tech Biopharmaceutical Co., Ltd. (Guangzhou, China). The EP, weighing 500 g, was soaked in 80% ethanol overnight, then subjected to two rounds of evaporative reflux (each lasting for 1 h) at 70 °C in a water bath. After that, the herbs were decocted with distilled water (10 times the volume), and the resulting mixture was extracted twice. The filtrate was combined, concentrated using rotary evaporation at 50 °C, and then centrifuged to obtain the supernatant. This supernatant underwent three cycles of alcoholic sedimentation before being freeze-dried to obtain the EPP sample. The glucose standard curve was measured according to the phenol-sulfuric acid method as y = 4.6767x + 0.1226, R^2^ = 0.9901. The sugar content of the extracted crude polysaccharide of EP was 45.76%, and the monosaccharide components of EPP were galacturonic acid (50.394%), galactose (19.844%), arabinose (16.123%), mannose (7.854%), and glucose (5.785%), which was identified by Wuhan Huaster Special Industrial Biotechnology Development Co., Ltd. (Wuhan, China) (Figure S1). Sodium dextran sulfate (DSS, molecular weight: 40,000 Da) was bought from Shanghai Kingbio Biosciences Co., Ltd. (Shanghai, China). The positive control drug sulfasalazine was provided by Shanghai Xinyi Tianping Pharmaceutical Co., Ltd. (Shanghai, China).

### 2.2. Animals Grouping and Modeling

Thirty-six six-week-old male Sprague–Dawley (SD) rats (specific pathogen-free) were supplied by the Guangdong Vital River Laboratory Animal Technology Co. (SCXK2022-0063, Guangzhou, China). The rats were raised at the Experimental Animal Center of South China Agricultural University (SYXK2022-0136, Guangzhou, China, 20 September 2022), which was carried out in compliance with national animal ethics regulations and supervised by the Animal Ethics Committee of South China Agricultural University (Protocol number: 2022b144). 

After one week of adaptation, the rats were randomly separated into six groups (*n* = 6): control (C), model (M), EPP low-dose (EPPL), EPP medium-dose (EPPM), EPP high-dose (EPPH), and the sulfasalazine (S) group. Rats in the C group drank pure water, while the other groups drank water with 4% DSS added. Six days were considered as one induction cycle, with a total of two cycles. After that, the treatment was administered intragastrically for 7 days, as follows: EPPL group (0.2 g/kg/d), EPPM group (0.5 g/kg/d), EPPH group (1 g/kg/d), and S group (0.1 g/kg/d); the appropriate amount of distilled water was administered to the other groups [20,22,23,24]. At the end of the experiment, rats were treated for cervical dislocation after anesthesia.

### 2.3. Clinical Index Observation

The disease activity index (DAI) score was assessed daily. The scoring criteria are shown in Table 1. The DAI score = (percentage of weight loss score + fecal viscosity score + fecal occult blood score)/3. The colonic length of each rat was measured.

### 2.4. Histopathology

The distal colon (about 1 cm) of rats was fixed in 4% paraformaldehyde solution for a week, and the colonic tissue was dehydrated, paraffin-embedded, sliced, and finally stained with hematoxylin and eosin (H&E).

### 2.5. Enzyme-Linked Immunosorbent Assay

Collected blood from the abdominal aorta of rats and centrifuged (3500 rpm/min, 15 min) to obtain the supernatant. The levels of interleukin-6 (IL-6), interleukin-1β (IL-1β), tumor necrosis factor-α (TNF-α), and transforming growth factor-β (TGF-β) were measured according to the instructions of the enzyme-linked immunosorbent assay (ELISA) kits (Yuanju, Shanghai, China).

### 2.6. RT-qPCR Analysis

We extracted total RNA from colon tissue and reversed transcript RNA to obtain cDNA. The procedure followed the reverse transcription kit instructions (Vazyme, Nanjing, China). Relative mRNA expression was calculated using the 2^−ΔΔCt^ method. Primer information is listed in Table S1.

### 2.7. Flow Cytometry Analysis

The rat spleen was grinded and filtered through a 40 μm cell sieve, and the spleen single cell suspension was prepared with a tissue lymphocyte isolation kit (Solarbio, Beijing, China). We collected rat MLNs, which were ground and filtered to obtain lymph node cells. For CD4^+^, CD25^+^, IL-17, and forkhead transcription factor protein 3 (Foxp3) staining, the cells were washed, fixed, and then stained using a flow cytometry staining kit (Elabscience, Wuhan, China). Sample loading detection was by Beckman (CYTOFLEX, Brea, CA, USA) flow cytometry and the results analysis used Flowjo V10 (Stanford, CA, USA).

### 2.8. Western Blot Analysis

Colon tissue was homogenized and centrifuged to collect the supernatant. Samples were put onto polyacrylamide gels with sodium dodecyl sulfate (SDS) for electrophoresis, after which the gels were shifted onto nitrocellulose blotting membranes. The membranes were blocked and incubated with anti-TLR4 (Bioss, Beijing, China), anti-myeloid differentiation factor 88 (MyD88) (Zenbio, Chengdu, China), and anti-nuclear factor kappa-B (NF-κB) p65 (Bioss, Beijing, China) at 4 °C overnight. Finally, we added horseradish peroxidase and incubated for 1 h [16]. The immunoreactive bands were detected with the ECL assay kit (Vazyme, Nanjing, China). Images were analyzed quantitatively using the Image J 1.53 software (Stuttgart, Germany).

### 2.9. Gut Microbiota Analysis

We extracted genomic DNA from fecal samples and quantified it. High quality genomic DNA samples were then used for PCR amplification. Primer 515F-806R was used to amplify the V4 region of the 16S rRNA gene [25].

### 2.10. Statistical Analysis

One-way analysis of variance (ANOVA) was employed to assess the differences between groups. Duncan’s new repolarization difference test and multiple comparisons were conducted using the least significant difference method (LSD). The *p*-values less than 0.05 were considered to be statistically significant differences between groups. Results were drawn using the Graphpad Prism 7.04 software (San Diego, CA, USA).

## 3. Results

### 3.1. EPP Improved Colitis Symptoms

**Figure 1:**
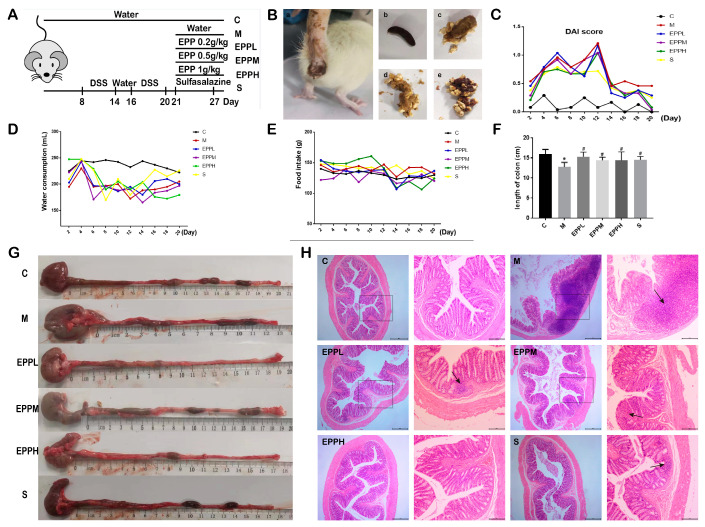
Therapeutic effect of EPP on rats with colitis. (**A**) Grouping and modeling of rats. (**B**) Different forms of rat feces. a. Diarrhea rats. b. Black, hard, shaped. c. Yellow, soft, half formed. d. Yellow, soft, shapeless. e. Blood fecal. (**C**) DAI score. (**D**) Changes in water consumption. (**E**) Changes in food intake. (**F**) Changes in colon length. (**G**) Measurement of colonic length in different groups. (**H**) H&E staining of colon tissue, observed at 40× and 100× magnification. * *p* < 0.05 vs. the control group. ^#^ *p* < 0.05 vs. the model group. The box shows the high magnification portion of the observation, and the arrow shows the lesion site.

In order to investigate whether EPP has an alleviating effect on colitis in rats, the DSS-induced colitis rats were treated with three different doses of EPP by gavage (Figure 1A). After drinking water combined with DSS, the rats exhibited symptoms, such as depression, loose stools, and an increase in the DAI index. The presence of blood in the stool and occult blood worsened as the modeling time increased (Figure 1B,C). Interestingly, it was found that although there was no significant difference in daily food intake among the rats throughout the process, there was a significant decrease in water intake during the modeling period for all groups except C group. However, this improved at the end of the modeling period (Figure 1D,E).

Compared with C group, the colon length of M group was significantly shortened (*p* < 0.05) and returned to the normal level after drug treatment (Figure 1F,G). As shown in Figure 1H, there was a large area of inflammatory cell infiltration, goblet cell deletion, and crypt deformation or deletion in the M group. EPP treatment showed obvious improvement, and there was only a small infiltration of inflammatory cells still present in the EPPL group and S group. A small amount of crypt loss was present in the EPPM group, and essentially no significant lesions were present in the EPPH group. Thus, the result indicated that EPP can confer a protection against DSS-induced colitis in rats.

### 3.2. EPP Restores the Th17/Treg Balance in Colitic Rats

**Figure 2:**
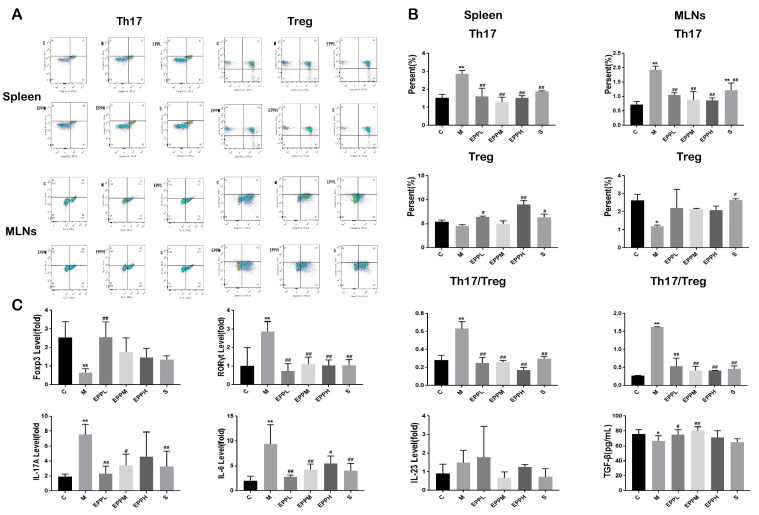
Effect of EPP on Th17/Treg balance. (**A**) Flow cytometry of the spleen and mesenteric lymph nodes. (**B**) Ratio of Th17 and Treg cells in the spleen and mesenteric lymph nodes. (**C**) Detection of genes and cytokines associated with Th17/Treg balance. * A significant difference compared with the control group, *p* < 0.05. ** A highly significant difference compared with the control group, *p* < 0.01. ^#^ A significant difference compared with the model group, *p* < 0.05. ^##^ A highly significant difference compared with the model group, *p* < 0.01.

The proportion of Th17 cells in DSS-treated rat spleen cells significantly increased (from 1.87% to 2.54%), while Treg cells showed a decreasing trend but no significant difference. Three doses of EPP treatment could reduce the proportion of Th17 cells in the spleen of colitis rats (*p* < 0.01), while EPPL and EPPH could significantly increase the frequency of Tregs (*p* < 0.05). The proportion of Th17 cells in DSS-treated rat MLN cells significantly increased (from 0.72% to 1.93%), while Treg cells were significantly reduced (*p* < 0.05). EPP treatment can significantly decrease (*p* < 0.01) the proportion of Th17 cells and the ratio of Th17/Treg (Figure 2A,B).

Meanwhile, the expression of RORγt and the levels of IL-17 and IL-23 were increased in the M group (*p* < 0.01). The level of RORγt could be significantly reduced after EPP administration (*p* < 0.01), The EPPL, EPPM, and S groups could significantly reduce IL-17 levels. On the contrary, Foxp3 expression, which is a Treg-specific transcription factor, and TGF-β were restored upon EPPL treatment, with other groups also showing an upward trend (Figure 2C). Therefore, it was concluded that EPP can protect colitis rats by regulating Th17/Treg balance.

### 3.3. EPP Regulated Inflammatory Response 

**Figure 3:**
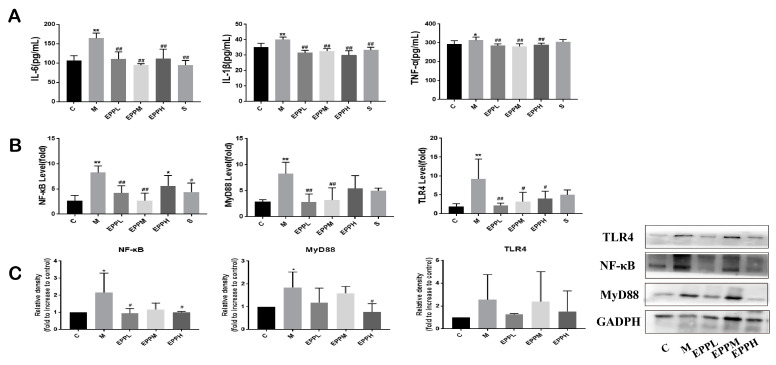
Detection of inflammatory factors and inflammatory signaling pathways. (**A**) Serum levels of inflammatory factors. (**B**) TLR4/MyD88/NF-κB signaling pathway detection by PCR. (**C**) TLR4/MyD88/NF-κB signaling pathway detection by western bolt. GAPDH is used as an internal control. * A significant difference compared with the control group, *p* < 0.05. ** A highly significant difference compared with the control group, *p* < 0.01. ^#^ A significant difference compared with the model group, *p* < 0.05. ^##^ A highly significant difference compared with the model group, *p* < 0.01.

As demonstrated in Figure 3A, the levels of IL-6 and IL-1β in serum were significantly increased in the M group (*p* < 0.01). However, the EPP in each dose group and the S group could significantly reduce this trend (*p* < 0.01). Additionally, the TNF-α level was significantly increased in the M group (*p* < 0.05), but EPP with each dose showed a significant alleviation of this trend (*p* < 0.01), even better than the S group. Exposure to DSS caused a significant increase in TLR4, MyD88, and NF-κB expression in the colon (*p* < 0.01), which was significantly reversed in both EPPL and EPPM groups. The EPPH group showed no significant difference in MyD88 and NF-κB expression compared to the M group but significantly reduced TLR4 levels were apparent (Figure 3B). The results of the Western blotting suggested that DSS induced an increase in the expression of TLR4, MyD88, and NF-κB proteins in the rat colon, and the expression of MyD88 and NF-κB were significantly different from the C group (*p* < 0.05). Compared with the M group, the EPPL group significantly downregulated the expression of NF-κB (*p* < 0.05), and the EPPH group significantly downregulated the expression of NF-κB and MyD88 (*p* < 0.05). All three doses of EPP were effective in inhibiting DSS-induced elevation of TLR4 expression (Figure 3C). Overall, EPP may achieve anti-inflammatory effects through suppressing the TLR4/MyD88/NF-κB signalling pathway.

### 3.4. EPP Regulates Intestinal Microbial Balance in Colitis Rats

**Figure 4:**
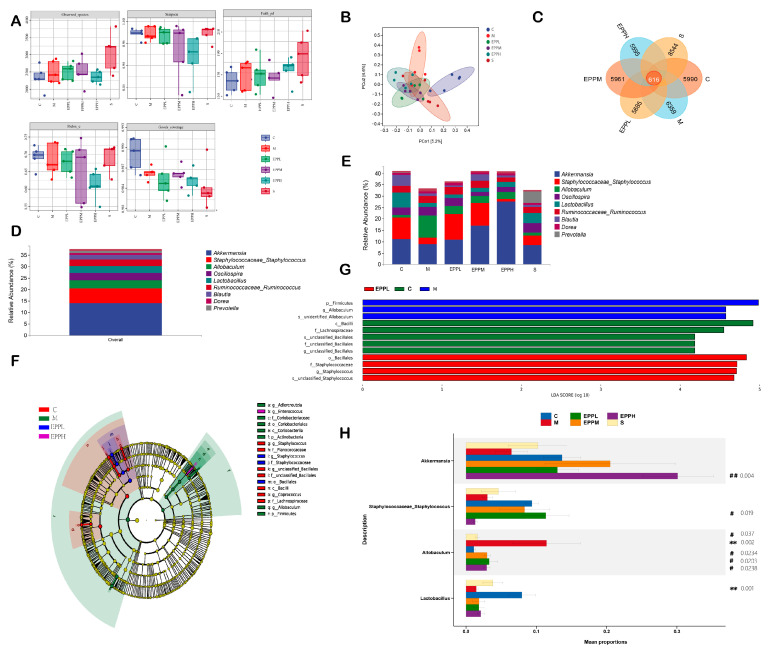
Effects of EPP on intestinal flora. (**A**) Alpha-diversity. (**B**) Beta-diversity. (**C**) Petal Venn diagram. (**D**,**E**) Flora composition. (**F**,**G**) Result of LEfSe. (**H**) Result of stamp. ** A highly significant difference compared with the control group, *p* < 0.01. ^#^ A significant difference compared with the model group, *p* < 0.05. ^##^ A highly significant difference compared with the model group, *p* < 0.01.

The differences in the gut microbiome are assumed to affect immune-associated diseases [26,27]. The observed species were presented for species richness and the Simpson index for species diversity. The PCA results in Figure 4B show that each group has obvious differences compared with the C group. There were 616 identical OTUs in each group (Figure 4A–C). The results showed that at the genus level, *Akkermansia* was the most abundant microflora detected in the rats used in this study. *Staphylococcus*, *Allobaculum*, *Oscillospira*, *Lactobacillus*, *Ruminococcus*, *Blautia*, *Dorea*, and *Prevotella* were also highly enriched. Compared with the C group, the proportions of *Akkermansia*, *Staphylococcus*, and *Lactobacillus* decreased in the M group, while the proportion of *Allobaculum* increased. Additionally, a dose-dependent increase in *Akkermansia* was observed after drug treatment in the EPP group. The *Staphylococcus* in the EPPL and EPPM groups was higher than that in the M group, while the *Allobaculum* in the EPPH group was lower than that in the M group; however, the *Lactobacillus* in the EPPH group was higher than that in the M group (Figure 4D,E). Different microbial genera among each group were analyzed by linear discriminant analysis combined with effect size measurement (LEfSe). *o_Bacillales* and *c_Bacilli* were significantly enriched in the C and EPPL groups, respectively, whereas g_*Allobaculum* was significantly enriched only in the M group (Figure 4F,G). Species-level analysis revealed that differences between different groups of bacteria, including *Akkermansia*, *Staphylococcus*, *Allobaculum*, and *Lactobacillus*, were different between the groups (Figure 4H).

## 4. Discussion

Polysaccharides are sugar chains formed by complex molecular structures bound by glycosidic bonds. Studies have indicated that polysaccharides are capable of altering intestinal immune homeostasis disturbance, maintaining the dynamic balance of inflammatory cytokines, and influencing CD4^+^ T cell development [28]. Some researchers have purified the total polysaccharides of EP and have finally isolated and named three purified EPPs, namely EPPS-1, EPPS-2, and EPPS-3, as well as demonstrating their anti-inflammatory and antioxidant effects [29]. In this experiment, we dried and crushed the whole herb of EP to obtain the crude polysaccharide through water extract–alcohol precipitation and lyophilization. The purification analysis of the crude polysaccharides of EP will be carried out in our subsequent experiments.

The model of chronic rat colitis was established using 4% DSS, which has been confirmed to be highly similar to human ulcerative colitis [30]. The pathological section of M group revealed that DSS caused extensive infiltration of inflammatory cells. However, EPP remarkably improved the pathological damage in the colon. Moreover, EPP can significantly reduce the levels of inflammatory cytokines in rats with colitis, especially in the medium-dose group, where it showed more excellent anti-inflammatory potential than the positive drug group. Combined with its effects on alleviating colonic length shortening and reducing DAI scores in rats with colitis, it can be concluded that gavage administration of EPP could alleviate colitis in rats. To facilitate sorting assays by flow cytometry, Treg cells can be labeled with CD4^+^, CD25^+^, and Foxp3, while Th17 cells can be labeled with CD4^+^ and IL-17 [31]. The result showed that EPP treatment significantly inhibited the production of Th17 cells in the spleen and suppressed Th17 cell production in the MLNs, while promoting the differentiation of DSS-suppressed Treg cells. It was shown that the expression of Foxp3 was significantly reduced in the M group but increased after administration of EPP, with a significant difference between the EPPL group and the M group. Meanwhile, EPP also significantly reduced the elevated expression of RORγt caused by DSS, which was consistent with the flow cytometry results. Thus, it can be concluded that EPP can regulate the Th17/Treg balance. Furthermore, cytokines also have an effect on T cell differentiation. Both Th17 and Treg cells use TGF-β as a signaling medium. Subsequently, when exposed to high concentrations of IL-6 or IL-21, naive CD4^+^ T cells can differentiate into Th17 cells. Some studies have shown that pro-inflammatory cytokines enhance this differentiation tendency [32,33]. The experiment showed that EPP treatment alleviates the decrease in TGF-β levels caused by DSS. The levels of IL-6, IL-1β, and TNF-α in serum from rats in the M group were significantly increased; however, EPP had an alleviating effect on these levels. According to some reports, the release of IL-23 with its association with IL-6 and TGF-β to stimulate the production of IL-23R is what actually makes Th17 cells harmful [34]. The experimental results showed an increasing trend in the gene expression of IL-23 that tended to increase in the M group, while the gene expression of IL-17A secreted by Th17 cells was significantly higher, and EPP could effectively inhibit this trend. Therefore, it is inferred that EPP can suppress the Th17/Treg imbalance induced by DSS by modulating cytokines.

TLR4/MyD88/NF-κB is a very classical signaling pathway closely associated with immunity and inflammation. TLR4 can signal through the MyD88-dependent signaling pathway, a critical control point for mediating signal molecular transduction. Upon TLR4 activation, MyD88 is recruited to activate NF-κB [35]. Activation of NF-κB triggers the overproduction of pro-inflammatory cytokines, such as TNF-α and IL-1β [36]. Results confirm a significant increase in the expression of TLR4, MyD88, and NF-κB in DSS-induced UC rats, and EPP effectively alleviated this trend, especially in the low-dose and medium-dose groups. Inhibition of the TLR4 pathway activation may be a feasible option for treating colitis, considering its detrimental impact on health. It was reported that macrophages possess Toll-like recognition receptors (TLRs) which can be activated by the bacterial product lipopolysaccharide (LPS). The stimulated macrophages exhibit different phenotypes [37]. It was interesting to note that some researchers [38] isolated and purified a novel homogeneous polysaccharide (EPPA) from Echinacea purpurea and found that it could enhance M1 macrophage polarization. The study may provide favorable support for the anti-inflammatory effects of EPP. However, there is still a great need for more studies to confirm whether EPP also affects macrophage polarization in the treatment of colitis.

Evidence is mounting that polysaccharides can act as prebiotics to assist in the maintenance of intestinal health [39]. The effect of DSS and EPP on intestinal flora of normal rats was obvious in the study. The sequencing results showed a greater proportion of *Akshmannia* and *Lactobacillus* in the control and EPP groups. However, contrary to the studies of others, significantly higher levels of *Allobaculum* were observed in the M group of rats. *Akkermansia* muciniphila is reported as being one of the most probable future-oriented intestinal probiotics [40]. It is interesting to note that several probiotics, including mucinophilic bacteria, have been demonstrated to have strain-specific impacts on the control of intestinal function. For example, therapy with *Lactobacillus casei* M2S01 successfully reduced the disease damage caused by DSS in mice but not the other *Lactobacillus casei* strains [41,42]. Intestinal flora may have an impact in the formation of Th17 cells, as evidenced by the finding that the number of Th17 cells in the mucus membrane of the intestine decreased considerably in mice after antibiotic therapy [43]. In general, there were inextricable relationships between intestinal flora and the inflammatory response and immune homeostasis, which need to be further explored to be fully understood.

The microbial–immune interaction theory of UC pathogenesis, which involves the appropriate balance regulation of anti-inflammatory and pro-inflammatory cytokines in intestinal mucosa to modulate epithelial barrier function, has received considerable focus [44]. Research has confirmed that EPP can alleviate colitis in rats by regulating Th17/Treg balance, suppressing the TLR4/MyD88/NF-κB signaling pathway, and modulating intestinal flora. In the current lack of specific drugs for UC, the therapeutic potential of EPP is promising. As a botanical ingredient, EPP not only enhances immunity but also offers the advantages of low toxicity and minimal side effects, providing new inspiration for the development of dietary supplements based on EPP. However, further research is needed for the isolation, purification, and structural characterization of EPP. Additionally, it is hoped that future studies will uncover more pharmacological effects of EPP.

## 5. Conclusions

In conclusion, the study demonstrated the therapeutic effect of EPP on DSS-induced colitis in rats. We observed that EPP as a dietary supplement could significantly improve the pathological changes in colonic tissues in rats with colitis, attenuate the shortening of colonic length induced by DSS, and reduce the levels of inflammatory factors in serum. This might be based on rectifying the Th17/Treg balance and suppressing the inflammatory pathway activation of TLR4/MyD88/NF-κB. In addition, EPP affected the diversity of intestinal microorganisms in rats, which might be a potential mechanism to gain more insight into the pathogenesis of UC and the immunomodulatory effects of EPP. Thus, we concluded that EPP has the potential to be used as a potential functional food ingredient for alleviating UC.

## Figures and Tables

**Table 1:** DAI scoring rules.

Score	Percentage of Weight Loss (%)	Fecal Viscosity	Fecal Occult Blood
0	<1	Black, hard, shaped	/
1	1~5	Black, soft and formed	Indicator is lilac
2	6~10	Yellow, soft, half formed	Indicator purple
3	11~20	Yellow, soft, shapeless	Indicator dark purple
4	>20	Yellow, watery stool	Blood fecal

## Data Availability

The data used to support the findings of this study can be made available by the corresponding author upon request.

## Supplementary Materials

The following supporting information can be downloaded at: https://www.mdpi.com/article/10.3390/foods12234265/s1, Table S1: Primer information used in the RT-qPCR; Table S2: Retention time of monosaccharide standards; Figure S1: Monosaccharide composition and molecular weight of EPP.
