# Supplementary material for: Echinacea purpurea (L.) Moench Polysaccharide Alleviates DSS-Induced Colitis in Rats by Restoring Th17/Treg Balance and Regulating Intestinal Flora
Source: Foods. 2023 Nov 25;12(23):4265. doi: 10.3390/foods12234265 (PMC10706068; doi:10.3390/foods12234265)
Supplement: Supplementary file 1 [file foods-12-04265-s001.zip › foods-2729582-supplementary.pdf]

Supplementary materials

Table S1 Primer information used in the RT-qPCR

Genes	Forward primer	Reverse primer
β-actin	TCAGGTCATCACTATCGGCAA	AGCACTGTGTTGGCATAGAGG
Foxp3	CCCTTTCACCTATGCCACCCT	TTGTGGCGGATGGCATTCTTC
RORγt	GAACCAGAACAGGGCTCAGAC	TAGAAGGTCCTCCAGGCGTAG
IL-17a	TGAAGGCAGCGGTACTCATC	GGGTGAAGTGGAACGGTTGA
IL-6	CTTCCAGCCAGTTGCCTTCTTG	TGGTCTGTTGTGGGTGGTATCC
IL-23	AAAAGTGACGTGCCCCGTAT	CAGACCTTGGCGGATCCTTT
NF-κB	TGTGGTGGAGGACTTGCTGAG	AGTGCTGCCTTGCTGTTCTTG
MyD88	CGACGCCTTCATCTGCTACTG	GCCGATAGTCTGTCTGTTCTAGTTG
TLR4	CTATCATCAGTGTATCGGTGGTCAG	ACAGCCAGCAATAAGTATCAGGTG

Table S2 Retention time of monosaccharide standards

Name	Abbreviations	Retention time
Mannose	Man	17.027
D-Galactosamine HCl	GlcN	20.167
Rhamnose	Rha	23.677
Glucuronic Acid	GlcUA	24.503
Galacturonic Acid	GalUA	28.123
D-Galactosamine HCl	GalN	32.215
Glucose	Glc	34.893
Galactose	Gal	39.578
Xylose	Xyl	41.732
Arabinose	Ara	43.442
Fucose	Fuc	50.422

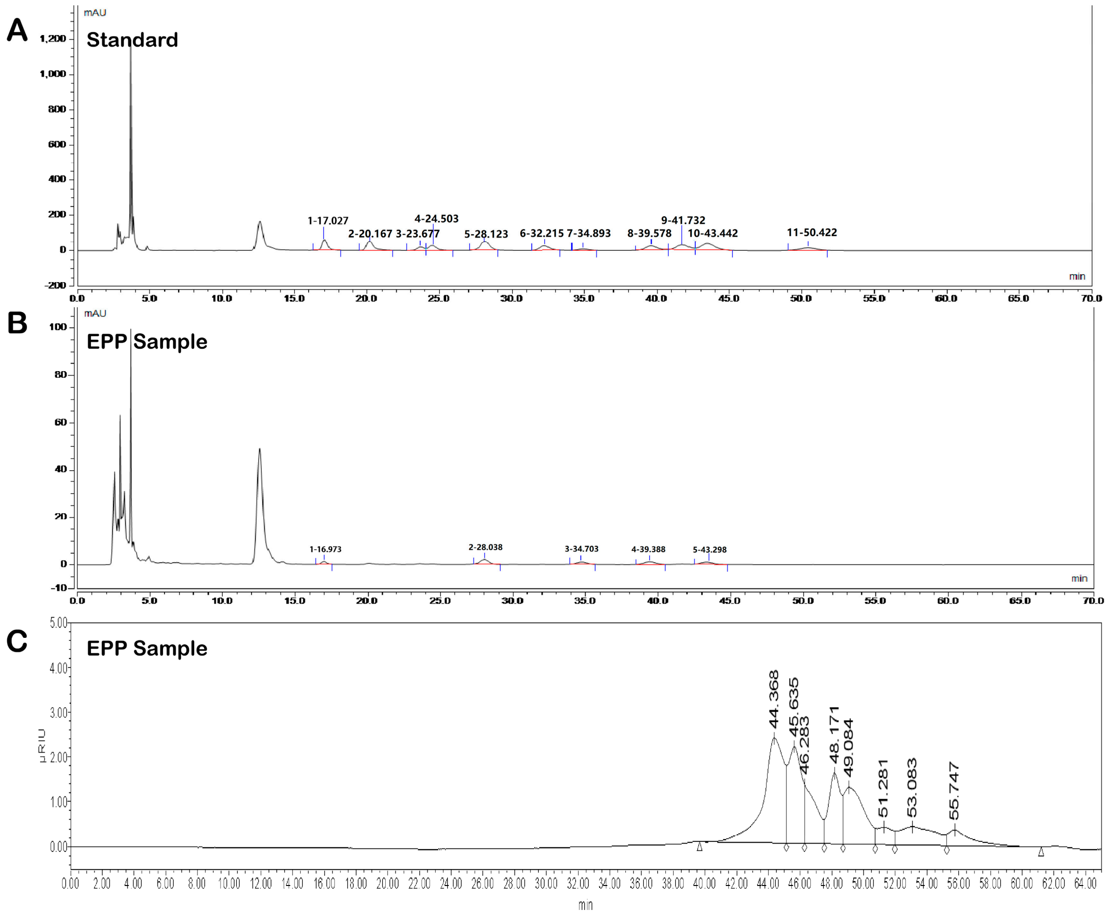

Figure S1. Determination of monosaccharide composition and molecular weight of EPP. (A)HPLC of monosaccharide standards. (B)HPLC of EPP sample. (C)HPGPC of EPP sample (The salt peak of mobile phase is near 48.2 min).
